# Characterization of the complete mitochondrial genome of Gangetic ailia, *Ailia coila* (Siluriformes: Ailiidae)

**DOI:** 10.1080/23802359.2019.1627942

**Published:** 2019-07-11

**Authors:** Md. Jobaidul Alam, Sapto Andriyono, Soo Rin Lee, Mostafa A. R. Hossain, A. T. M. Eunus, Md. Tawheed Hassan, Hyun-Woo Kim

**Affiliations:** a Interdisciplinary Program of Biomedical, Department Mechanical and Electrical Engineering, Pukyong National University, Busan, Republic of Korea;; b Fisheries and Marine Faculty, Universitas Airlangga, Surabaya, Indonesia;; c Department of Fisheries Biology & Genetics, Bangladesh Agricultural University, Mymensingh, Bangladesh;; d WorldFish, Dhaka, Bangladesh;; e Department of Aquaculture, Sylhet Agricultural University, Sylhet, Bangladesh;; f Department of Marine Biology, Pukyong National University, Busan, Republic of Korea

**Keywords:** Next-generation sequencing, *Ailia coila*, mitochondrial genome, Bangladesh

## Abstract

The first complete mitochondrial genome sequence of *Ailia coila* from Bangladesh was determined by the bioinformatic assembly of the next generation sequencing (NGS) reads. The constructed circular mitogenome for *A. coila* was 16,565 bp in length which harbored the canonical 13 protein-coding genes, 22 tRNAs, 2 rRNAs. Two non-coding regions, control region, *D-loop* (927 bp), and origin of light strand replication, O_L_ (30 bp) were also well conserved in the mitogenome. Among the currently reported mitochondrial genomes in the order Siluriformes, *A. coila* was most closely related to *Eutropiichthys vacha* (AB919123) with 85.63% sequence identity.

## Introduction

The Gangetic ailia, *Ailia coila* is an important freshwater small indigenous species (SIS) in Bangladesh, India, Pakistan, and Nepal. Although it has been commonly found in rivers, and lakes, the habitats of *A. coila* are sharply decreasing due to the various anthropogenic and natural causes (Afsar 1990) and this species is currently categorized as vulnerable species in Bangladesh and India (Khan et al. 2000; Mijkherjee et al. 2002; Hanif et al. 2015). It is now urgently required to conserve its genetic information for the sustainable use of the resources in those countries. We here report the first complete mitochondrial genome sequence of *A. coila*, which was collected from Bangladesh.

The specimen was collected from Sylhet, Bangladesh (24°40′12″ N, 91°49′59.88″ E) in March 2017 and the species identification was confirmed by both the morphological characteristics and the sequence identity in COI region in the database (KT364761). The specimen is stored at the Department of Fisheries Biology & Genetics laboratory, Bangladesh Agricultural University, Mymensingh-2202, Bangladesh, and Department of Marine Biology, Pukyong National University, South Korea. The mitochondrial DNA was extracted with a commercially available kit (Abcam, USA) and fragmentation of the mitochondrial DNA was conducted by Covaris M220 Focused-Ultrasonicator (Covaris Inc., USA). A library was constructed by TruSeq^®^ RNA library preparation kit V2 (Illumina, USA) and its quality and quantity was analyzed by 2100 Bioanalyzer (Agilent Technologies, USA). DNA sequence of *A. coila* was determined by the Illumina MiSeq sequencer (2 × 300 bp pair ends) and the obtained raw reads were further assembled by Geneious software ver 11.0.2 (Kearse et al. 2012). The secondary structures of tRNA were predicted by the ARWEN program (Laslett and Canbäck 2008) and the phylogenetic tree was constructed by MEGA7 using the Minimum Evolution (ME) algorithm (Kumar et al. 2016).

The complete circular mitogenome of *A. coila* (MK348534) was 16,565 bp in length, which consisted of 13 protein-coding genes, 22 tRNAs, and 2 ribosomal RNAs (12S and 16S). Two non-coding regions including a control region (927 bp), and the origin of light strand replication (O_L_) were also well conserved in its mitogenome. The control region was identified between *tRNA-Pro* and *tRNA-Phe*, while the O_L_ was between *tRNA-Asn* and *tRNA-Cys* at the WANCY tRNA cluster. The overall A + T content of the *A. coila* mitogenome was 59.10% and its gene arrangement was identical to other Siluriform fish. All the other 21 tRNAs were predicted to be folded into the typical clover-leaf structures, except for the tRNA^Ser^. Similar to the other Silurid catfish (Alam et al. 2019), an unusual start codon was exclusively identified in *COX1* gene (GTG). Incomplete stop codons (TA−/T–) were predicted in 6 genes including *ND2*, *COX2*, *COX3*, *ND3*, *ND4*, and *CYTb* genes.

**Figure 1.**
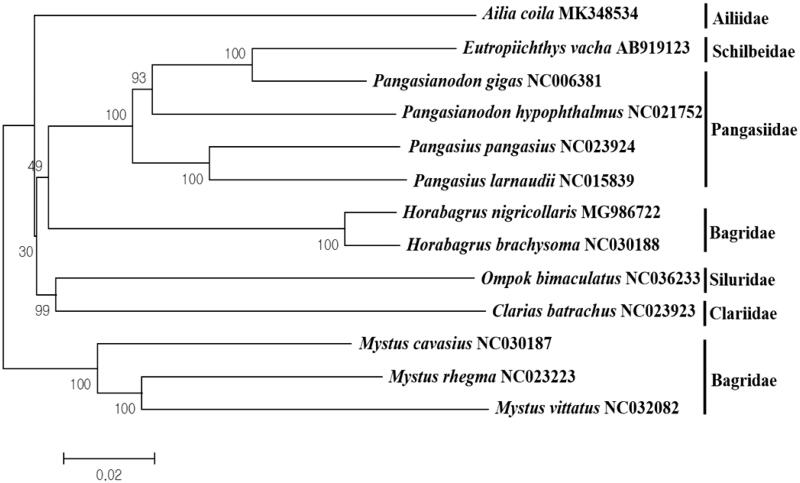
Phylogenetic relationship of *Ailia coila* in the order Siluriformes. Phylogenetic tree was constructed with the currently reported complete mitogenomes in the order Siluriformes using MEGA7 software using the Minimum Evolution (ME) algorithm with 1000 bootstrap replications. GenBank accession numbers are shown followed by each species scientific name.

As the only species in the genus *Ailia* (Nelson 1994; Froese and Pauly 2010), *A. coila* formed a different clade from other Silurid species (Figure 1). Among the currently reported mitogenomes in the order Siluriformes, *A. coila* was most closely related to *Eutropiichthys vacha* (AB919123) with 85.63% sequence identity.
